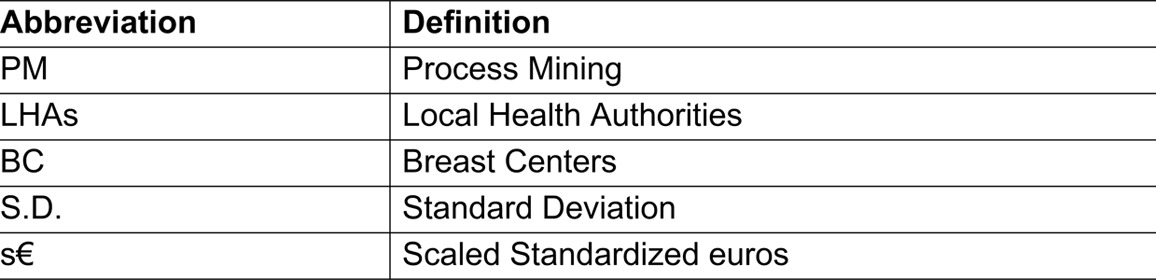# Enhancing quality and decision-making for care pathways: An application of process mining in cancer care

**DOI:** 10.1371/journal.pone.0339788

**Published:** 2026-01-07

**Authors:** Francesca Ferré, Chiara Seghieri, Sima Sarv Ahrabi, Andrea Burattin, Andrea Vandin

**Affiliations:** 1 Institute of Management, Management and Health Lab, L’EMbeDS, Sant’Anna School of Advanced Studies, Pisa, Italy; 2 Department of Biomedical Sciences for Health, University of Milan, Milan, Italy; 3 DTU Technical University of Denmark, Kgs. Lyngby, Denmark; 4 Institute of Economics, L’EMbeDS, Sant’Anna School of Advanced Studies, Pisa, Italy; Makere University College of Health Sciences, UGANDA

## Abstract

Care pathways are widely used as evidence-based clinical governance tools to enhance the quality of care of groups of patients with a specific clinical problem and optimize the use of resources. However, it is often the case that there are differences between the recommended care pathway and the actual clinical practice. Recently, Process Mining (PM) techniques, a family of data-driven techniques from computer science that uses logs (execution traces) of a system to reason about its underlying process, have been applied in the healthcare context to map and analyze real-world practice patterns. In particular, PM helps to discover and analyze the sequence of activities, to highlight variances and possible sub-optimal management of the clinical paths in order to improve care quality and reduce the inefficient allocation of resources. Using the breast cancer pathway as a case study example, this study aims to describe the application of PM to administrative healthcare data of public hospitals of the Tuscany Region (Italy) and to offer insights about strengths and limitations in data management, information creation, and interpretation to support decision-making. The study revealed variations in the management of breast cancer care pathways across different public healthcare providers and with respect to the recommended guidelines. Key findings include instances of service duplication, delays, and bottlenecks, particularly in the diagnostic phase. The analysis also highlighted variations in healthcare costs, driven by differences in the frequency and types of diagnostic exams or visits performed. The findings have practical implications for enhancing the efficiency and quality of breast cancer care and provide a practical example of how the methodology can be applied to other healthcare contexts for similar benefits.

## 1 Background

### 1.1 Introduction

A care pathway is a complex intervention for the organization of healthcare assistance to groups of patients with a specific clinical problem that needs integrated and multidisciplinary care across different settings (such as outpatient, hospital, and follow-up). Care pathways are used as clinical governance tools to enhance the quality of care of groups of patients with a predictable clinical course and optimize the use of resources [1–3]. They are multidisciplinary management tools based on the best available evidence-based practice in which the different tasks (interventions) by the professionals involved in patient care are defined, optimized, and sequenced, and outcomes are tied to specific interventions. They are used to structure and design care processes and improve them within the patient-centered care concept [4]. A recent review of the literature highlights several benefits, including patient centrality, a clearer definition of roles and responsibilities among professionals, improved clinical decision-making, an increased integration of care processes with embedded coordination structures, dynamic learning, and adaptability to physical, social, and resource constraints [5]. However, it is often the case that there are differences between the care pathway designed and the actual clinical practice. There can be several reasons for this, e.g., best practice guidelines could not be present or could represent idealized scenarios that do not reflect reality [6] or do not reflect patients’ preferences [7]. In addition, such differences may be caused by organizational factors leading to long queues of patients waiting for visits or exams, or bottlenecks in care settings where actions that could be done in parallel occur sequentially. Analyzing and examining healthcare processes can help improve care pathways and patient treatment [8]. To do so, it is essential to understand the current state of the practices [9]. Once discrepancies between real-world and intended practices are identified, they can be used to support quality improvement initiatives and feed into the performance management cycle. Over the years, several analytical data-driven approaches have been proposed to shed light on this complex domain. A recently applied and promising approach is the so-called *process mining* (PM) [10]. The main idea is to exploit *logs* on the execution of a system to *reverse engineer* the system by obtaining a compact and understandable representation of how a system actually behaves as opposed to how it is expected to behave (as prescribed by a *reference* process). In general, methods such as PM help organizations manage their processes in a more informed and effective manner [10] and provide evidence for quality improvements [11]. Although PM originated primarily within the industrial domain, it has been more recently applied to social systems. For example, international *initiatives* [12] have been created to direct efforts in applying PM to the healthcare context and to promote methodological developments to make PM fit better for this domain [13]. Using PM to map and analyze real-world practice patterns (the actual process) and exploring deviations from deliberated care pathways (the reference process) can help identify sub-optimal management of the clinical path, improve care quality, and reduce the inefficient allocation of resources. Well-recognized and quite common care pathways suitable for this application are the oncological clinical pathways, such as the breast cancer one, where evidence-based clinical guidelines are available and the condition trajectory is quite predictable. Indeed, breast cancer is a common disease with a relatively good prognosis: it is the most common cancer diagnosed in women, and approximately 80% of women living in OECD countries are alive 5 years after diagnosis [14]. The management of breast cancer is complex and is best performed within the context of a specialist multidisciplinary breast center to ensure optimal clinical outcomes [15]. In the last decade, internationally recognized quality assurance clinical guidelines have been developed, promoted, and used to monitor the quality of breast cancer care [16,17] along all phases of breast cancer care, including screening, diagnosis, and treatment. Given these premises, using the breast cancer pathway as a case study example, this study aims to describe the application of PM to real-life healthcare data, performing a comparative analysis across public hospitals to gain richer information about the care pathway performance, and offering insights about strengths and limitations in information creation and interpretation to support decision-making and enhance process-level results. To the best of our knowledge, this study will be the first to apply PM to administrative data, analyzing and comparing care processes and costs within healthcare operations of several providers in a large regional healthcare system in Italy.

### 1.2 Related work

Care pathways are healthcare processes characterized by a dual complexity: they are complex due to the multiple interacting components within the pathway itself and the need to navigate the external and internal complexities of the organizations in which they are implemented [18–20]. Management scholars have heavily studied and reported approaches to continuously improve process management and enhance customer value through optimizing operations (i.e., minimizing waste) with the application of “lean” ideas in healthcare facilities and by reducing variation in service organizations by decreasing defects to a specific statistical measure (Six Sigma) or applying the combination of the two (i.e., Lean Six Sigma) [21–23]. The implementation and assessment of quality management practices is traditionally developed through case studies supported by quantitative and qualitative data collection and analysis, aiming at understanding the flow of patients, supplies, or information through the journey of a ‘standard’ patient [24,25]. In some instances, the availability of recognized healthcare quality indicators allows for the continuous assessment of key performance metrics along the care pathway, considering specific process and outcome indicators, which can be represented in a benchmark [26]. In more recent years, the emergence of analytical data-driven approaches has enabled the use of PM to systematically explore and understand healthcare processes. Indeed, an increasing number of studies provide literature reviews on PM in healthcare topics from a variety of perspectives [13,27–32]. Recent reviews further confirm the growing methodological and clinical relevance of PM across medical domains. For instance, Chen et al. [33] systematically examined PM and data mining applications in chronic disease management, while Kusuma et al. [34] reviewed PM studies on disease trajectories. In oncology, Kurniati et al. [35] provided an early and influential overview of PM applications, illustrating the potential of these methods to uncover variations in cancer care pathways. These works collectively demonstrate the maturity of PM research in healthcare and support the rationale for its application to breast cancer care pathways in our study.

A comprehensive review that is based on PM methods and other data-driven methods to determine the actual patient care pathways can be found in the work by [36] and [37]. The results suggested that the explored methods deliver valuable information for healthcare planning and management. In the paper by [38], PM techniques are exploited in the healthcare system to support service reconfiguration. Their results support decision-makers by analyzing patient flow and estimating the resources needed for specific patient groups. An overview of common use cases of PM in healthcare research and its adoption challenges is discussed in [39]. For example, [40] uses PM to understand patient satisfaction and assist in its improvement. Differently, predictive models based on de facto pathways were developed by [41] to predict waiting times. [42] analyzed pathways from a physical position perspective and applied a constrained Markov Reward Process to optimize patient waiting times. The application of data-driven process simulation in healthcare is proposed by [43] based on a Belgian hospital’s radiology department activities. A detailed analysis of the several necessary steps from preprocessing of data to the application of a set of different PM algorithms to explore processes with a focus on waiting times for patients in an Egyptian hospital can be found in [44]. By utilizing PM techniques, a new method for discovering dynamic risk models for chronic diseases is introduced in [45]. Differently, [46] discusses the potential outcomes of using standard clinical coding schemes to describe event log data and classify medical terminology. A recent unified view of the characteristics of PM and an overview of key challenges of that for healthcare, such as methodologies and frameworks, conformance checking, and attention to data quality, are discussed in [47]. The authors of [48] have examined the role of clinical guidelines and medical protocols in healthcare, highlighting the field’s fragmentation and its impact on developing universally accepted solutions. The study by [8] proposes improvement to PM usability and understanding. When narrowing down to research in the area of PM in cancer care, we found an active research community. A process-analytic approach studies the system during patient chemotherapy treatment using clinical records [49]. A conformance analysis of translated clinical guidelines in oncology is conducted by [50] using the pMineR library. Few experiences are reported in the literature to address PM in breast cancer. A study conducted by [51] establishes empirical relationships between patient flow problems, healthcare service quality, and patient satisfaction in emergency departments, using a mixed-method research approach and partial least squares structural equation modeling (PLS-SEM). Chen et al. [52] identify differentially expressed genes in triple-negative breast cancer and explore the key pathways. An application of PM to breast cancer and its efficacy, along with preliminary results, is discussed by [53]. Table 1 summarizes some articles in the literature related to process mining approaches for breast cancer data.

**Table 1:** A synoptic overview of the main related work on process mining in breast cancer with various perspectives.

Ref.	Scope of study	Observation
[54]	Perform careflow mining and phenotype identification	University of Pavia and the Salvatore Maugeri Foundation
[49]	Evaluate statistical and clinical approaches	Breast cancer - Leeds Cancer Centre
[55]	Apply a careflow mining algorithm	Hospital Maugeri of Pavia, Italy
[56]	Obtain a Markov model for patient pathways during chemotherapy	UK oncology centre
[52]	Explore the key pathways and genes of TNBC	Gene Expression Omnibus Datasets
[51]	Investigate the relationship between pathways and the cost	Swedish breast cancer quality registry and case-costing system
[57]	Compare processes for different patient populations	Hospital Group Twente database, Netherlands
[58]	Analyze the sequence of actions	public healthcare system of the Basque Country, Spain
[53]	Preliminary results on application of PM to breast cancer data	public healthcare system of Tuscany region, Italy

## 2 Methods

### 2.1 Organization and management of breast cancer in Tuscany

**Fig 1:**
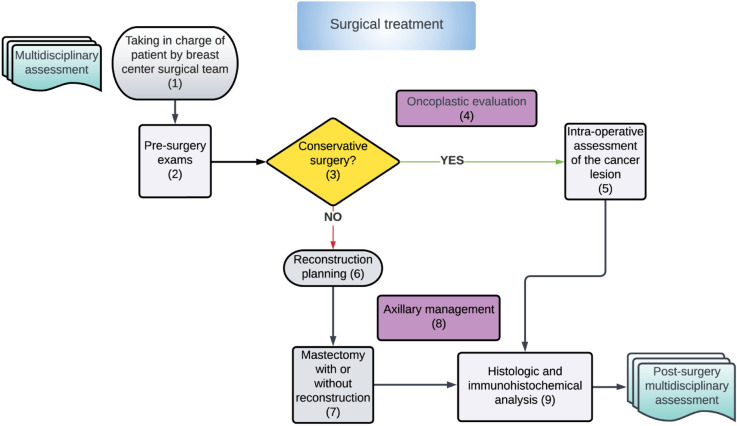
Regional Decree 3823/2019, Tuscany region.

This study focuses on Tuscany, a large region in central Italy (over 3.7 million inhabitants, about 6.2% of the Italian population) characterized by the provision of inpatient services almost exclusively through public providers (with over 95% of all hospitalizations provided by public hospitals), in a non-competitive system in which patients are free to choose where to receive care. The regional healthcare system comprises three local health authorities (LHAs) with 48 district general hospitals directly managed by the LHAs, four teaching hospitals, and 26 health districts, which oversee the organization and delivery of services for local health networks. Each LHA has a large catchment area (about 90 0000 resident population). Hospital care is reimbursed using Diagnostic-Related Group tariffs, although this method is generally complemented with other forms of payments, such as global budgets for specific care services, e.g., emergency and hospital teaching activities. The tariff model is not applied to hospitals run directly by LHAs, which are reimbursed through a capitation system. The Tuscan health system is highly centralized, and its main goal is to reduce unwarranted variations among hospitals and health districts to increase value for the patients and the population. The Tuscany healthcare system is considered one of the best-performing regional health systems in Italy, and well-developed in cancer care [59]. Improving cancer care remains a high priority in the healthcare agenda of the regional service, where complex interventions are required to ensure high-quality and equitable access to diagnostics, treatment, and follow-up services. Clinical guidelines on oncological care are provided through the regional comprehensive cancer care network. The cancer network includes all cancer departments (six) and is a governance tool aiming at increasing continuity of care (integration of otherwise separate care services), reducing variability, and enhancing clinical research and knowledge diffusion. The cancer network provides a collaborative space for the coordination and integration of multidisciplinary actors along the cancer care pathway. Within the cancer network, a specific breast cancer network is active counting on 14 Breast Centers (BC). A BC is an equipped multidisciplinary and multi-professional breast clinic where breast cancer is diagnosed and treated. Thus, a place or network of providers where all the services necessary, from genetics and prevention through the treatment of the primary tumor to care of advanced disease, palliation, and survivorship are available [60]. Despite this comprehensive definition, Tuscany BC is traditionally identified with hospital units where treatments are provided, including surgical treatments, chemotherapy, and radiotherapy. Indeed, requirements focus on the availability of a multidisciplinary team approach with a clinical coordinator, a dedicated radiologist, a dedicated breast surgeon and breast pathologist, a medical oncologist, and a radiotherapist. Additionally, a minimum caseload of 150 newly diagnosed cases of primary breast cancer to be treated each year by each provider is required [60]. To standardize clinical practice and streamline coordination among professionals in breast cancer care, a breast cancer regional care pathway has been defined based on evidence-based clinical guidelines developed by national and international scientific societies (reference Regional Decree 3823/2019). This clinical governance tool defines the typical patient pathway across services and treatments with a clear indication of the operational sequence (spatial and temporal) of services and activities provided for taking charge of women with breast cancer from screening to post-surgical treatment. They identify the responsibility and interdependence between professionals, units, and organizations. For our study, we focus on the path from cyto-histological diagnosis to surgical treatment, considering both malignant breast cancer and localized malignant breast cancer (in situ). Women with a diagnosis of breast cancer are included in this pathway, and a full pre-surgical diagnostic assessment is necessary for tumor characterization and a correct indication of surgical intervention. Moreover, the guideline states that the time interval between the end of the diagnosis and the surgery itself has to be no more than 30 days for surgery with the highest surgical priority. Fig 1 displays a schematic representation of the guideline.

### 2.2 Data preparation

In this study, we used individual-level administrative data from the Tuscany region between January 1, 2018, and December 31, 2018. We accessed the database in May 2019. The Tuscany healthcare administrative databases contain information regarding all public and private accredited healthcare providers. Specifically, this study used two data sources: (i) hospital inpatient data; (ii) outpatient care data. The data are pseudo-anonymized at the regional health information system office, where each patient is assigned a unique identifier (Patient ID) that applies to all administrative databases. This identifier does not include the patient’s identity or other sensitive data. We included only women resident in the Tuscany region who were hospitalized with a principal or secondary diagnosis of malignant breast cancer and had a mastectomy, conservative or reconstructive surgery. We use the International Classification of Diseases, $9^{\text{th}}$ Revision, Clinical Modification (ICD-9-CM) codes 174 (malignant breast cancer), 233.0 (localized malignant breast cancer—in situ), and intervention codes 85.2x (conservative surgery), 85.33, 85.34, 85.35, 85.36 (reconstructive surgery), and 85.4.*x* (mastectomy). Our cohort of interest is women with elective surgery with the highest surgical priority without age restrictions.

**Data extraction.** Before creating a process map, i.e., a graphical representation of the *actual* process followed by Tuscan breast cancer patients, features must be selected and cleaned.

Surgical interventions have been classified according to the type of surgical approach into simple mastectomy, reconstruction post-mastectomy, and breast-conserving therapy. However, in the note, we refer to them as “surgery.” Briefly, we followed a conservative approach by focusing on patients’ records containing highly reliable data, discarding those that might be less reliable or complete. The patients were excluded if:

- Lived outside Tuscany or within Tuscany but underwent surgery outside the regional healthcare system. For these patients, we might miss some information regarding treatments outside Tuscany.
- Did not have any record regarding their priority of surgery. This is because the priority specifies the temporal constraints to be followed.
- Have not had at least one mammogram or ultrasound within one year before the surgery. Likewise, if they did not have a biopsy. From domain knowledge, it is known that patients undergoing one of the considered surgeries should have had such exams; missing information might be due to diagnostic exams taken outside the Tuscany region or in a private setting.
- The elapsed time between diagnosis and surgery exceeds 365 days (considered as outliers).
- They had more than one surgery. We prefer to focus on patients with one surgery only, as patients necessitating more surgeries are likely to follow paths adjusted case-by-case.

Having removed such patients, cleaning up unnecessary activities is essential to avoid creating an unnecessarily complex process map. Records contain three types of activities: diagnostic exams like mammography and biopsy, pre-surgery exams like electrocardiography, and follow-up exams after surgery. This classification has been done automatically based on the date of the surgery as opposed to those of such exams. A biopsy involves removing small pieces of breast tissue from the suspected area for evaluation in a pathology lab. A biopsy can only determine whether the tumor is benign or malignant [61]. After a positive biopsy (malignant tumor), acute care typically involves several time-sensitive interventions. The guidelines recommend quick referral to treatment. For example, women with the highest priority indication for surgery are expected to receive intervention within 30 days after diagnosis, as included in the National Plan for managing waiting times 2019–2021 of the Italian Ministry of Health [62]. In our analysis, it was not possible to identify the exact date of diagnosis; therefore, we considered the date of the last breast biopsy as the starting point of the analyzed surgical pathway. The endpoint was defined as the date of surgery. This time window (from biopsy to surgery) ensures a consistent and clinically meaningful process segment across all patients. From a methodological perspective, this choice also supports the Fuzzy Miner algorithm implemented in Disco, which benefits from well-defined start and end events to produce coherent and interpretable process models. Including activities outside this window (e.g., pre-diagnostic exams or post-surgery follow-up) would have introduced heterogeneity and reduced model clarity. Pandas [63], one of the most popular Python packages for data processing, is used to clean the data in this part. In total, the prepared data for analysis include 2245 patients with 10906 events and 7 types of activities (the different exams, the different surgeries, etc.)

**Ethics approval and consent to participate:** The results reported in this paper were obtained from healthcare administrative data of the Tuscany region (Italy). The authors have access privileges to the data under an agreement signed between Scuola Superiore Sant’Anna of Pisa and the regional administration—Direzione Diritti di Cittadinanza e Coesione Sociale - of Tuscany (Italy) for supporting the regional bodies in the performance evaluation of the healthcare system. Ethical approval for this study was waived by The Joint Ethical Committee for Research of the Scuola Superiore Sant’Anna and the Scuola Normale Superiore on December 2024 (Delibera n.63/2024).

### 2.3 Tools and techniques for Process Mining

In PM, step-by-step events can be represented graphically, enabling domain experts and decision-makers to visualize and reason about the actual processes running in the considered system. In healthcare PM, the best techniques are those that are able to handle a large amount of noise and enable the sorting of individual behaviors for separate analyses [64]. The health process models in this work are discovered by using the Fuzzy Miner algorithm, a refined implementation of which is supported by the commercial tool Fluxicon Disco [65]. The descriptions and procedures are based on the Disco version 3.3.7. The tool allows for a deeper understanding of complex (healthcare) models, also thanks to interactive techniques to reduce the complexity of mined processes by coarse-graining the information contained in them. Each patient is assigned a unique identifier (Patient ID), which labels all events regarding activities involving him/her and the date and time of each event. The Fuzzy Miner algorithm, implemented in Fluxicon Disco, was selected because this study aimed to achieve an intuitive and high-level understanding of care pathway behavior rather than an exact control-flow reconstruction. Fuzzy Miner dynamically abstracts complex and noisy event logs, emphasizing the most frequent and relevant paths while aggregating infrequent ones. This makes it particularly suitable for administrative healthcare data, which often contain heterogeneous, incomplete, or low-frequency events. Other process discovery algorithms, such as the Heuristic Miner or the Inductive Miner, are more appropriate for producing precise control-flow models, but they tend to generate highly complex or fragmented models in this context. Compared to more recent approaches (e.g., [34,66]), which integrate event attributes or transition features, our approach prioritizes interpretability and visual simplicity for supporting managerial and clinical decision-making. This choice aligns with the study’s goal of highlighting key practice variations, bottlenecks, and cost differences across hospitals rather than constructing executable models.

Furthermore, the log files can contain optional information that may be necessary to evaluate a process [65]. In particular, in our study, we also have information about the number and type of diagnostic exams, visits, and their associated cost expressed through tariffs. To establish a process map, we select the extracted features required for investigation from biopsy to surgery, as discussed in Sect 2.2.

**Fig 2:**
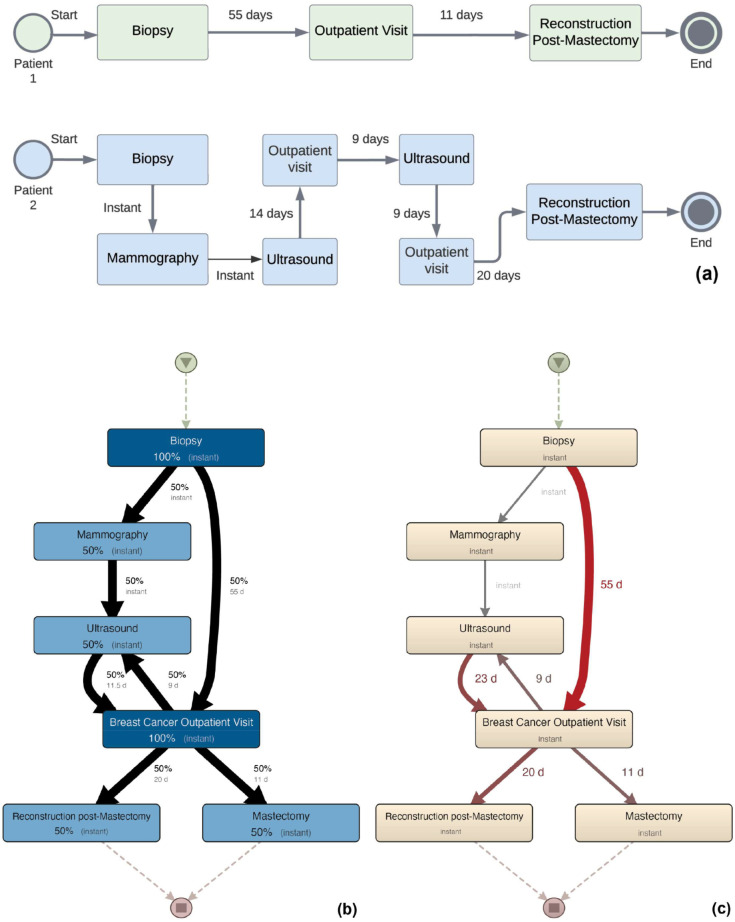
Event logs and waiting times with corresponding process maps, extracted from biopsy to surgery for two hypothetical patients (excluding non-diagnostic exams). (a) shows the two patients’ care pathways. (b) displays the frequency view of the process map mined from panel (a). (c) presents the performance view of the same process.

**Fig 3:**
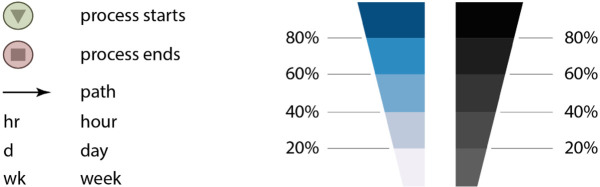
Legend of the process maps.

**Structure of the process map.** Fig 2, panel a, denotes the activities of two hypothetical patients. They undergo a biopsy and an outpatient visit at the beginning and middle of the process, respectively. Upon experiencing a biopsy, patient 1 was examined by a physician specialist and then sent to surgery. Instead, patient 2 had a more complex journey from biopsy to surgery. We now discuss the structure and visual representation of a process map, based on Fig 2, panels b and c, which depict the process maps mined for the two patients. In particular, Fig 2, panels b and c, provide two different *views* for the same mined map: the former, *frequency view*, focuses on frequencies of paths and events (i.e., the case coverage), while the latter, *performance view*, focuses on temporal aspects. We provide only once, in Fig 3, the legend for all the maps in this paper describing their graphical notation.

**Boxes and arrows.** The activities involving a patient are represented as boxes, while arrows indicate the process flow between them (i.e., we have an arrow between two activities if at least one patient does the two activities in sequence without any intermediate ones). To create paths (or pathways) from the beginning to the end of a process, the boxes are arranged following the temporal order of the corresponding activities, from earliest to latest (top to bottom), when possible. For example, consistently with the two patients in Fig 2, panel a, the two maps in Fig 2, panels b and c, show that biopsies always come before mammograms.

**Weights, thickness, color intensity, and dashes.** Fig 2, panels b and c, also shows that boxes and arrows are labeled with weights and quantitative information like frequencies and elapsed time, enabling one to visually spot frequent paths and bottlenecks. This information is also given graphically, leveraging the thickness of the arrows and the color intensity of the activities. In particular, in Fig 2, panel b, thickness and color intensity denote the frequency of each arrow and node (e.g., how many patients do a certain activity), while in Fig 2, panel c, they denote the average elapsed time. For example, Fig 2, panel a, shows that only patient 1 directly does an outpatient visit after a biopsy, after 55 days, the longest elapsed time between any two activities. Consistently, we can see a 50% label and a 55*d* label, respectively, for the corresponding arrow in Fig 2, panels b and c, with the latter arrow being the thickest one showing the longest time. In Fig 2, panel c, all boxes are labeled with “instant.” This is because our data does not contain the start and end times of the activities, but only the day. Therefore, this label shall be ignored in our study. Instead, the instant label of arrows signifies the completion of both the source and target activity on the same day. Dashed arrows point to activities that occurred at the very beginning or the very end of the process. This can allow spotting *deadlocks*, i.e., unexpected early terminations of treatments.

**Importance zoom.** One of the primary outcomes of process mining maps is to collect, highlight, and reveal in a compact way the many pathways taken by the users, with the possibility to filter for the most common or uncommon ones. In other words, a decision-maker might be interested in only the most dominant paths and activities in the process map or the least common ones (the variations). Disco allows tuning this by offering “importance zooming” capabilities in the form of a slider for both activities (boxes) and transitions (paths in the maps), and it makes sure that no depicted activity is disconnected in the obtained *zoomed* map. For the maps in Fig 2, we set both zoom sliders to 100%, meaning all activities are visualized. However, for the process maps in Sect 3 generated from real data, we found that setting such a zoom slider to 80% for the paths (essentially, for the arrows) is a good trade-off between the completeness and interpretability of the models.

#### 2.3.1 Configuration of the frequency and performance views in our study.

As discussed, Disco features two map representations, or *views*, to focus on frequencies of events and paths (frequency view) or on time performances of the processes (performance view). These can be configured in several ways. We now discuss the setting used in our study.

**Frequency view.** This view, exemplified in Fig 2, panel b, reports *weights* (and corresponding arrows thickness and color intensity) on how frequently an activity (a box) or a sequence of two activities (an arrow) is in the logs. This view is particularly useful because it allows us to spot the least and most frequent paths. In our analysis in Sect 3, we do not consider absolute frequencies but percentiles of the number of patients in each breast cancer hospital to understand and compare the process maps obtained for different hospitals. This is because we consider patients at hospitals with a heterogeneous number of served patients.

**Performance view.** This view, exemplified in Fig 2, panel c, reports *weights* connected to temporal aspects. The view can be configured to report several performance metrics for activities and paths, like, e.g., the total/mean/median time spent by all patients in moving from biopsy to mammography. Among the various performance metrics offered by Disco, we find that the median is particularly well-suited for this study. Indeed, the median is important to mitigate the impact of outliers. Since patients undergo a variety of procedures, some of which are more complex than others, a median value would indicate typical performance. This perspective is especially valuable, as it enables the identification of performance bottlenecks.

**Mixed frequency/performance view.** As a matter of fact, Disco allows for the compression of information from the two views into a single map. Indeed, Fig 2, panel b, actually provides performance information. By looking at the labels of arrows, we can see two pieces of quantitative information. The main one, with larger fonts, is frequency information, while the secondary one, with smaller fonts, provides performance information. In fact, Disco allows one to enrich a view with secondary information from the other using a smaller font. Thicknesses and color intensities from another view cannot be added. In all maps presented in Sect 3, we will provide this compact mixed view with the two discussed frequency and performance information.

## 3 Results

### 3.1 Preliminary discussion

In total, after applying our data cleaning, we found 10 906 events that accounted for 2245 patients hospitalized in 19 hospitals of the Tuscany Region with an overall median case duration of 53 days. We grouped all recorded events for the studied patients by the hospital where they had the surgery. Given the high number of Tuscan hospitals and their heterogeneity, as an example, we present the results of four hospitals; results for all Tuscan hospitals are available upon request. We consider two hospitals among those with high volumes of breast cancer patients and two among those with low volumes. Hospitals are sorted from the highest number of treated patients to the lowest and assigned anonymized names H1, H2, H3, and H4 for high, median, and low numbers of surgeries. The findings have been presented and discussed with breast cancer surgeons of the hospitals included in the analysis during working meetings conducted for the tuning of data and their interpretation. Table 2 displays some basic information about women hospitalized with breast cancer. Since the data used in this study does not contain explicit information about the day of tumor diagnosis, we use the date of the first biopsy as a proxy. Indeed, biopsies are central to the diagnostic process of (breast) cancers. In other words, case duration reported in Table 2 considers the time interval from *first* biopsy to surgery.

**Table 2:** Statistics on patients who underwent surgery in the four hospitals considered. Case duration is calculated from the time of the first biopsy to the time of surgery.

Hospital	N. patients	N. events	Avg. events per patient	Median case duration (days)	Total cases
H1	≥250	1871	3.6	51	23%
H2	≥250	2858	5.8	65	22%
H3	150–249	1069	4.8	47	9%
H4	<150	281	3.6	53	3%

From Table 2, we see that the four selected hospitals are heterogeneous in the number of cases treated, of recorded events, and of per-patient events. We also provide the median duration of cases and the percentage of cases handled by each hospital over the total regional cases. We can see that the hospital with the largest case duration has the highest average number of events per patient. However, by looking at the other hospitals, we do not see a clear pattern relating the average number of events per patient and case duration. In this section, we use PM techniques to shed more light on this. We apply Disco (Fuzzy) Miner to discover and visualize the processes of patients according to the surgery hospitals. As discussed in Sect 2.3, we set the *importance zooming* for paths and boxes to 80% and 100%, respectively.

**Fig 4:**
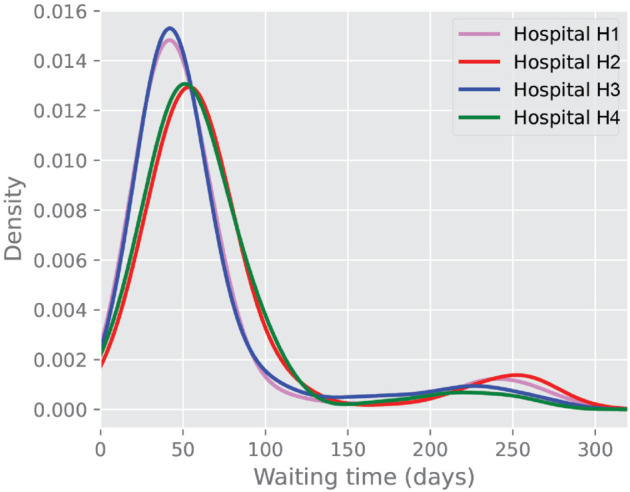
Waiting time distribution: Case duration (biopsy to surgery) for each hospital.

We aim to analyse the elapsed time for the preoperative phase at the hospital level. To this end, we compare the distribution of elapsed time from the first biopsy exam to surgery for each hospital. Fig 4 seems to indicate a pairwise similar pattern in hospitals H1 and H3, as well as H2 and H4, although with some differences across them. In particular, median waiting times from biopsy to surgery for the four hospitals were 55 (IQR: 25-130) days for H1, 57 (IQR: 42-76) days for H2, 44 (IQR: 35-58), and 52 (IQR: 40-73) days for H3 and H4, respectively. Together with waiting times and volumes, we also included measures of the costs of diagnostic exams across the different hospitals. In particular, for costs, we computed direct healthcare costs obtained from the regional tariffs and performed some variation analysis across hospitals. We used standardized costs (in euros) to ensure comparability across different hospitals. The procedure involved calculating the cost associated with each diagnostic exam, factoring in the exam’s frequency and its unique tariff. Indeed, it is possible that some exams can be performed differently, reflecting different approaches or diagnostic technologies used, thus producing variations in costs across hospitals. For instance, within the context of biopsy, four different approaches are reported, and they reflect different procedures: from the simplest fine needle biopsy to stereotactic breast biopsy, which includes X-ray technologies. Choices are based on the radiologist’s judgment and reflect the range of available resources in the hospital.

### 3.2 Hospital H1

Hospital H1 is a teaching hospital in a metropolitan area with a breast center performing over 840 surgeries a year.

**Fig 5:**
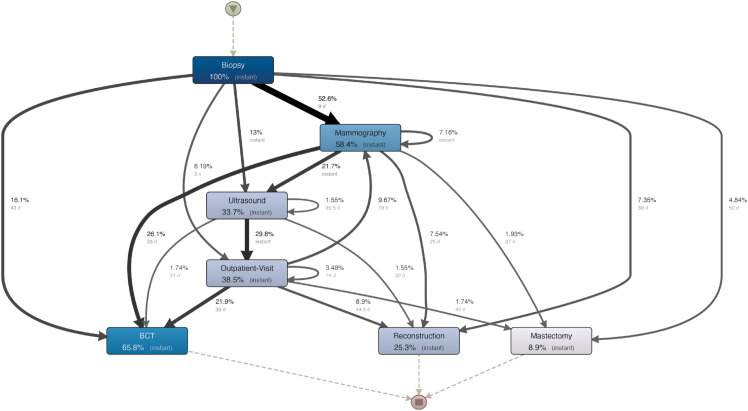
Process map mined for hospital H1.

**Process map.** The map extracted by Disco for patients from this hospital is provided in Fig 5. In all cases, the process starts with a biopsy, performs combinations of visits (mammography, ultrasound, outpatient visit), and then ends up undergoing one of the three considered types of surgery. In particular, most of the patients (about 66%) undergo conservative surgery. We analyze the paths and activities with the most participants, i.e., those involving the thickest arrows and the boxes with the darkest colors, as well as the median time (the secondary quantitative information given in smaller font) between activities to identify where the delays occur. According to the map, the median waiting times for all patients who directly proceeded from biopsy to reconstruction, biopsy to mastectomy, and biopsy to BCT were 39, 50, and 43 days, respectively. Also, we note that ultrasound exams tend to happen right after biopsy or mammography (the *instant* label in the corresponding arrows denotes exams done on the same day). We also see that all exam nodes have *self-loops*, meaning that patients might do the same exams several times. For example, mammography tends to be repeated on the same day in 7.16% of cases. In reviewing the surgeries and their associated earlier activities, it is evident that except for 26.1% of patients treated immediately after mammography in a median time frame of 23 days, the remaining flows did not conclude before 25 days, and in some cases, not until 37 days. In about 3.5% of the cases, we had repetitions of breast cancer outpatient visits, with a median elapsed time of 14 days. Table 3 reports a summary of the costs of exams (direct costs obtained from regional tariffs associated with each exam) and variation information at the hospital level. The table considers the costs per patient in euros (s€). Additionally, this table provides information on the number of “types,” which indicate the various types of examinations performed at the hospital. Finally, the column frequency of the tables specifies the proportion obtained by dividing the number of exams performed in a hospital by the number of treated patients. We can see that:

- **Biopsy**: H1 has a median cost per patient of 0.71 s€, a mean cost per patient of 12.11 s€, and a standard deviation (S.D.) of 19.57 s€. We have frequency 1 because, as discussed, we consider only patients who have had at least one biopsy; thus, the entire population considered has received a biopsy. We find a variation count (types) of 4, meaning that the hospital performed 4 variants of biopsies, specifically stereotactic breast biopsy with retroaspiration, stereotactic breast microbiopsy, echo-guided breast biopsy with retroaspiration, and echo-guided fine-needle breast biopsy.
- **Ultrasound**: We found a median cost of 1.70 s€ per patient, a mean cost of 4.23 s€ per patient, and an S.D. of 4.65 s€. The frequency of 0.41 indicates that less than half of the patients underwent this examination, and a Type 2 includes monolateral or bilateral ultrasound.
- **Mammography**: The hospital recorded a median cost of 2.36 s€ per patient, a mean cost of 4.77 s€ per patient, and an S.D. of 7.41 s€. The frequency of 0.71 implies that a majority of patients underwent this examination. We found 2 variants of this exam. Again, it could refer to monolateral mammography or bilateral mammography.
- **Visits**: For visits, we find a median cost of 5.43 s€ per patient, a mean cost of 5.43 s€ per patient, and an S.D. of 7.06 scaled euros. The frequency of 0.5 indicates that about half of the patients underwent this examination. We found 2 variants of this exam, one coded as a first breast visit and the other as a follow-up breast visit.

**Table 3:** Costs and volume of exams conducted at hospital H1. Costs are expressed in scaled standardized euros (s€). The table reports the median, mean, and standard deviation (S.D.) of costs, along with the frequency and number of types of each exam.

	Median	Mean	S.D.	Frequency	Types
**Biopsy**	0.71	12.11	19.57	1.00	4
**Ultrasound**	1.70	4.23	4.65	0.41	2
**Mammography**	2.36	4.77	7.41	0.71	2
**Visits**	5.43	5.43	7.06	0.50	2

### 3.3 Hospital H2

Hospital H2 is a teaching hospital with a breast unit performing over 700 surgical interventions per year.

**Fig 6:**
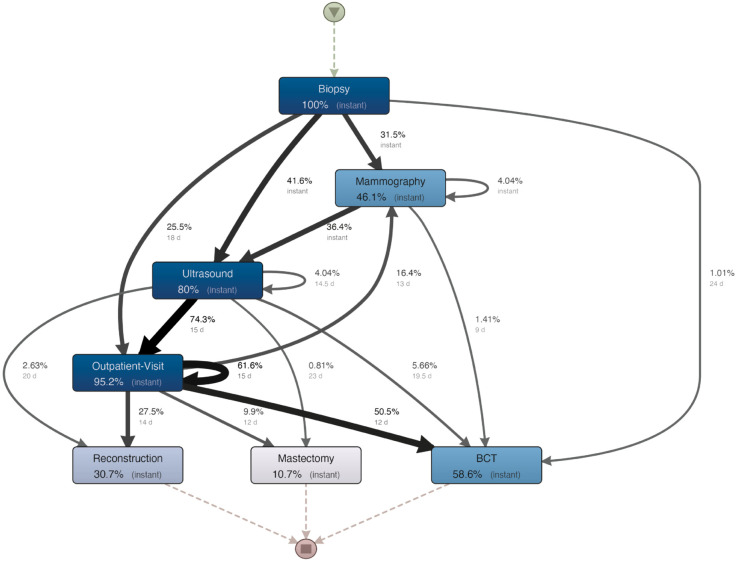
Process map mined for hospital H2.

**Process map.** Fig 6 displays the process model. The map shows that at least 31% of the women had their mammography and ultrasound done quickly. To identify bottlenecks, we again focus on paths and activities with the most participants. According to Fig 6, breast visits and ultrasounds are the most frequent activities. The two activities are connected by a frequent path taken by two-thirds (74.3%) of the patients, with a median time between them of 15 days. Furthermore, the process model shows repetitions for breast visits for 61.6% of patients, with a median waiting time of 15 days. In addition to this, 16.4% of women have a (possibly repeated) mammography after a breast visit.

Table 4 includes a summary of examination cost and variation data at the hospital level:

- **Biopsy**: Patients underwent a biopsy with a median cost per patient of 1.01 s€, a mean cost per patient of 8.25 s€, and a standard deviation of 17.52 s€. As for H1, we found 4 variations.
- **Ultrasound**: Patients incurred a median cost of 1.37 s€ per patient, a mean cost of 11.72 s€ per patient, and an S.D. of 18 s€. The frequency of 1.02 suggests that the majority of patients underwent this examination, with some repetitions. As for H1, we found 2 variations.
- **Mammography**: The hospital recorded a median cost of 1.83 s€ per patient, a mean cost of 3.58 s€ per patient, and an S.D. of 5.42 s€. The frequency of 0.54 indicates that about half of the patients underwent this examination. In H2, we found one more variation than in H1 related to tomosynthesis, a three-dimensional mammogram.
- **Outpatient visits**: For visits, we found a median cost of 24.21 s€ per patient, a mean cost of 24.21 s€ per patient, and an S.D. of 33.95 s€. The frequency of 2.20 indicates that multiple visits occurred per patient. We found only one variant for this exam.

**Table 4:** Costs and volume of exams conducted at hospital H2. Costs are expressed in scaled standardized euros (s€). The table reports the median, mean, and standard deviation (S.D.) of costs, along with the frequency and number of types of each exam.

	Median	Mean	S.D.	Frequency	Types
**Biopsy**	1.01	8.25	17.52	1.00	4
**Ultrasound**	1.37	11.72	18.00	1.02	2
**Mammography**	1.83	3.58	5.42	0.54	3
**Visits**	24.21	24.21	33.95	2.20	1

### 3.4 Hospital H3

Hospital H3 performs approximately 350 surgical interventions each year.

**Fig 7:**
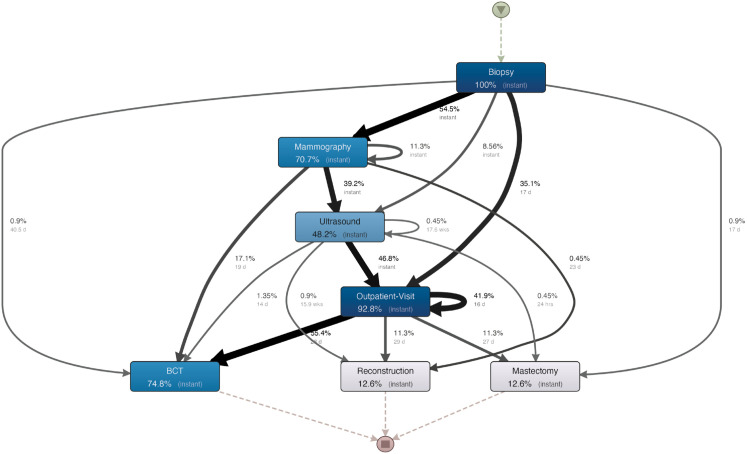
Process map mined for hospital H3.

**Process map.** A breast visit is the most common activity before surgery included at this hospital, with 92.8% of cases covered (Fig 7). There is a longer median elapsed time between a breast visit and surgery in this hospital than in the previous one. The process before the visit, however, has been relatively fast. Additionally, we have repetitions of visits with a median waiting time of 16 days, which might be considered a bottleneck in the process. This hospital experiences a much higher percentage of BCT surgeries than the previous two. Table 5 summarizes the information on costs and variation of exams at H3:

- **Biopsy**: Patients underwent biopsies with costs per patient that had a median of 2.23 s€, a mean of 22.76 s€, and a standard deviation of 53.37 s€. As for H1 and H2, we found 4 variations.
- **Ultrasound**: Patients had ultrasounds with a median cost per patient of 2.47 s€, a mean of 5.00 s€, and an S.D. of 4.37 s€. About half of the patients underwent this examination, with 4 variations.
- **Mammography**: The hospital reported mammography costs with a median of 5.45 s€ per patient, a mean of 5.24 s€ per patient, and an S.D. of 4.42 s€. A significant proportion of patients participated in this examination, and there were 3 variations (the three-dimensional mammogram was used).
- **Outpatient Visits**: Visits were associated with median costs of 16.12 s€ per patient, a mean of 16.12 s€ per patient, and an S.D. of 22.32 s€. Multiple visits were made by patients for 2 variations of the exam.

**Table 5:** Costs and volume of exams at hospital H3. Costs are in scaled standardized euros (s€).

	Median	Mean	S.D.	Frequency	Types
**Biopsy**	2.23	22.76	53.37	1.00	4
**Ultrasound**	2.47	5.00	4.37	0.50	4
**Mammography**	5.45	5.24	4.42	0.84	3
**Visits**	16.12	16.12	22.32	1.47	2

### 3.5 Hospital H4

In a given year, the hospital H4 performs approximately 190 surgical interventions.

**Fig 8:**
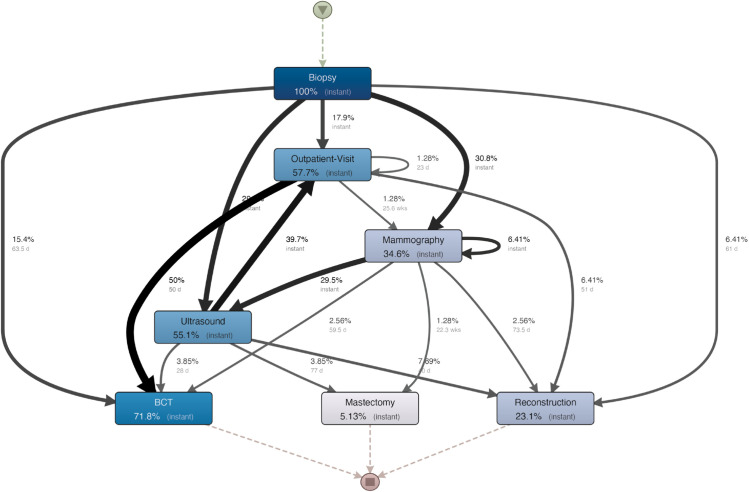
Process map mined for hospital H4.

**Process map.** The process map computed for this hospital is shown in Fig 8. Notably, all the arrows entering surgeries have considerably high waiting times. In particular, there is a median elapsed time of 50 days between breast visits and BCT, which is performed for 50% of patients. Instead, there has been a reasonably rapid flow between different diagnostic examinations. Finally, a notable percentage of patients (22%) had a particularly short process, having surgery right after a biopsy (15.4% had a BCT, while 6.41% had a reconstruction). However, this did not lead to fast treatment, as for such patients we found a median time larger than 60 days (63.5 days for BCT and 61 days for reconstruction). It is interesting to notice that, among the four considered hospitals, only H1 has a relevant fraction of patients (20.94%) with such short processes, i.e., biopsies followed directly by surgery. Instead, hospitals H2 and H3 only have a negligible percentage of such patients. This analysis allows us to conclude that the operation of this hospital could be improved by reducing the elapsed time from the last exam to the surgery.

We now move our attention to the costs and variations of exams in hospital H4, as given in Table 6:

- **Biopsy**: Hospital H4 conducted biopsies with median costs of 2.84 s€ per patient, mean costs of 20.10 s€ per patient, and a standard deviation of 40.70 s€. As for all the other hospitals, we found 4 variations of this exam.
- **Ultrasound**: We found that ultrasound examinations incurred median costs of 0.56 s€ per patient, mean costs of 6.89 s€ per patient, and an S.D. of 11.21 s€. About half of the patients had such an examination, with 2 variations.
- **Mammography**: The hospital reported mammography costs with median expenses of 1.78 s€ per patient, mean expenses of 2.76 s€ per patient, and an S.D. of 2.90 s€. Around 42% of patients had this examination of 4 variations.
- **Outpatient Visits**: Visits at hospital H4 were associated with median costs of 12.97 s€ per patient, mean costs of 12.97 s€ per patient, and 0 standard deviations. About half of the patients had outpatient visits, with no variation (the code used is the first breast visit only).

**Table 6:** Costs and volume of exams at hospital H4. Costs are in scaled standardized euros (s€).

	Median	Mean	S.D.	Frequency	Types
**Biopsy**	2.84	20.10	40.70	1.00	4
**Ultrasound**	0.56	6.89	11.21	0.59	2
**Mammography**	1.78	2.76	2.90	0.42	4
**Visits**	12.97	12.97	0	0.59	1

## 4 Discussion

In recent decades, the introduction and adoption of care pathways across Europe have significantly increased, aiming at guiding evidence-based care. These pathways define the optimal care processes, specifying timing, sequencing, and types of care based on the best available evidence, thereby contributing to the promotion of care quality and improved efficiency. However, despite their widespread use, there remains a pressing need for real-world studies that record and analyze potential challenges and gaps in the implementation and effectiveness of care pathways in routine clinical practice. Traditional performance information systems, though effective in monitoring key aspects of care pathways and identifying red flags for system comparisons, are insufficient at the operational level , because they lack an understanding of the determinants of performance. To bridge the gap between real-world practice and the expected performance of care pathways, additional data insights are needed on the hospital floor. As reported by the literature, conformance checking techniques are frequently employed to compare process models with event logs [50], and the results of the analysis can produce a descriptive picture (frequency and/or performance view) or rather provide the basis for investigating the root causes of potential discrepancies that may exist. For example, the descriptive picture supports clinicians and healthcare managers in timely identifying and highlighting deviations in the patients’ journey from the recommended pathway in terms of timing, number, types of interventions, sequencing, etc., and also differences in the use of resources and thus differences in healthcare costs.

**Fig 9:**
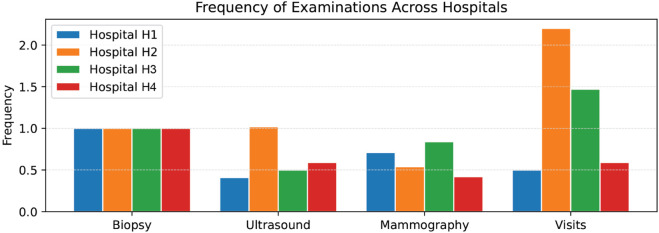
Frequencies of average examination types across four hospitals.

**Fig 10:**
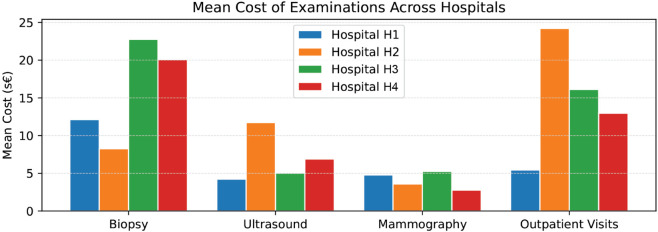
Costs of average examination types across four hospitals, in scaled standardized euros (s€).

**Fig 11:**
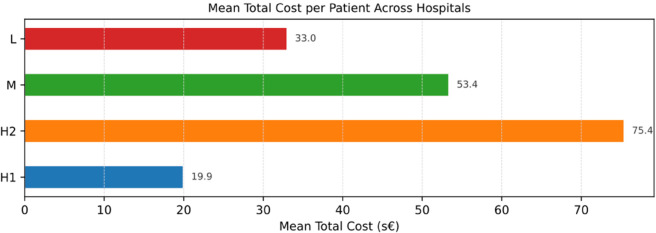
Mean total cost per patient, obtained by combining the average cost and frequency of each examination type for the diagnostic phase (biopsy–surgery), in scaled standardized euros (s€).

In our study, we have focused on a specific section of the breast cancer care pathway and have highlighted differences both in the patient process and related costs across multiple hospitals. We have employed a comparative process analysis, allowing us to observe differences in patient pathways across different organizations, providing insights into an approach that is still underexploited. The comparative analysis of PM indicates that hospitals employ analogous diagnostic methodologies for women with suspected breast cancer, including mammograms, ultrasounds, and consultations with a breast surgeon prior to surgery. However, the frequency and timeliness of access do not always follow a single path; there are repetitions of exams or jumps that make the cases different from the others and dissimilar from the suggested care pathway. Fig 9 provides a comparison of the average number of pre-surgical exams across hospitals. Also, in terms of healthcare costs, the PM analytical approach can help describe differences at the hospital level. For example, despite the uniform frequencies of biopsies shown in Fig 9, their costs vary significantly (Fig 10). H3 has the highest mean cost per patient, followed by H4, H1, and H2. In our analysis, these variations seem to largely depend on the different diagnostic approaches and technologies available in the hospitals. As a result, while frequency and cost are related, they do not move in parallel. Finally, Fig 11 summarizes the estimated mean total cost per patient, obtained by combining the average cost and frequency of each examination type. This figure provides an overall view of the diagnostic expenditure, ranging from about 20 s€ in Hospital H1 to more than 75 s€ in Hospital H2, with intermediate values for Hospital M (53 s€) and Hospital L (33 s€). One significant driver in variation is the use of different diagnostic technologies (biopsy, for instance) that have a direct impact on costs and also the timing of the care path, since more advanced techniques can reduce the repetition of the exam and increase the accuracy and timeliness of the diagnosis. Further investigation would be beneficial to understand the underlying reasons for these variations, especially in times of personalized medicine, where genomic testing and genetic profiling will be routinely included in the diagnostic phase. However, the normative approach to the analysis is of foremost importance to understand the nature of such discrepancies. Indeed, deviations may be either positive or negative. They may be beneficial if they enable patients to advance efficiently toward the outcome as a result of determinants of illness or patient preferences. They might be negative when variations are not driven by patients’ needs and preferences but are deviations from the dictates of evidence-based medicine [7].

Care pathways should be flexible to accommodate potential differences in patients’ needs. To this end, process mining shows overall process events at a glance and is able to immediately identify and quantify the frequency of local evidence-practice gaps. In this study, by exploiting the advantages of process mining techniques in healthcare, we depict the processes of breast cancer patients in and across four representative public hospitals of the Tuscany region as a case study example. The application of process mining techniques provided visual maps of the patient processes across services and time. The analysis supports the identification of service duplication, service delays, constraints, and bottlenecks specific to each hospital site. For example, the analysis revealed instances of longer waits during the diagnostics phase in some hospitals compared to others due to longer elapsed time between examinations. This evidence can be informative to hospital management to support improvements in streamlining the care process by changing the allocation of resources or the priorities or by improving efficiency. Furthermore, the evidence suggests exam repetitions that should be carefully examined to understand their appropriateness. As shown in Figs 9—11, hospitals presented relevant differences in diagnostic intensity and overall expenditure per patient. The variations observed in the mean total cost (Fig 11) may reflect heterogeneity in clinical practices and resource allocation, suggesting opportunities to harmonize procedures and optimize efficiency across hospitals. The PM data visualization and data interpretation are additional valuable elements to support quality improvement actions at the organizational level, together with the traditional performance monitoring systems already in place. Indeed, the complexity of the healthcare system and the complexity of the care pathway pose significant challenges to ensuring effectiveness in care pathway implementation [20]. It appears clear that the combined use of performance and analytical techniques, which are interdependent, can help in understanding the complexity of the system when analyzed together. This exploratory study has some limitations. First, it would benefit from the inclusion of other endpoints of interest, not only waiting times and costs but also clinical outcomes and other information regarding, for instance, the date of the diagnosis, the use of healthcare personnel along the pathway, which is not available in administrative care data. Second, data quality (e.g., coding of events) and completeness may undermine the comparative analysis. This seems to be a common concern reported by the literature despite the advancement in information systems [67]. Third, when many cases deviate, the process map becomes hard to read; although process mining tools give a simple overview, the detailed view should be interpreted with support from domain experts. Indeed, analysis of the causes of deviation is not immediately identifiable, and the necessary interpretations and verification of organizational processes in the field are necessary. For this reason, in this study, the design and output of the analysis were shared among clinicians to make them aware of the variations in the everyday process and discuss the potential causes of these variations to reflect on actions needed to make the care journey more effective. The literature emphasizes the need to continuously engage clinical stakeholders, both for interpretation and to support the effective use of findings in clinical governance and reporting and communication to a broader audience [67]. More generally, PM analyses are always presented as tasks that require continuous iterations and that should be embedded into the BPM lifecycle of the organization adopting it [10,68]. In addition, while some parts of the PM analysis can, in principle, benefit from some level of automation, the interpretation phase and the identification of actionable insights remain primarily human activities (by domain experts). In a broader perspective, the findings of this study contribute to the growing international evidence on the potential of process mining for improving healthcare delivery. Healthcare processes are inherently complex and context-dependent, and deviations from standard pathways are not always negative but can reflect necessary clinical flexibility or patient-centered adaptations. Future research should aim to refine process mining techniques to better accommodate such complexity and enhance their applicability across diverse healthcare systems and settings. We can envision future PM-centric research along two dimensions. On the one hand, PM activities include many more tasks beyond the discovery of the process maps presented here. For example, conformance checking [69] or model repair [70] can be useful techniques to, respectively, verify the adherence of patients with clinical guidelines and to suggest possible adjustments to the guidelines themselves. On the other hand, if data becomes available as a continuous stream, then all the analyses can be performed in real-time via *streaming process mining* [71], thus providing up-to-date information regarding the status of patients, thus giving the possibility of monitoring and managing the patients according to clinical guidelines and patients’ needs and preferences. It is important to emphasize that the latter still requires fundamental PM research and is not yet fully developed.

## 5 Conclusion

In this study, we present an application of PM tools to analyze breast cancer care across hospitals in a large Italian region, using data from 2018 as an example. At the regional level, a performance management system supports the planning, organization, and evaluation of various healthcare activities, including cancer care [72]. This system facilitates benchmarking across local health authorities and hospitals based on key performance indicators, demonstrating how the region has progressively improved its cancer care outcomes, ultimately positioning itself as a top performer at the national level. However, while this tool is effective for benchmarking performance, it is less suited for providing operational insights into patient pathways or assessing the concordance with established care pathways. Overall, the systematic benchmark of care pathways across care providers through the application of PM techniques provides useful insights for variability management, process reengineering can be used as a clinical audit tool. This tool allows for the constant revision and redesign of care processes according to best practice and aids the improvement of guidelines based on real-world evidence. A key strength of this study is the ability to compare data across hospitals, providing critical perspectives on variation management. How this variability can be explained is of primary importance for future work. Experts should invest in the identification of measures that evaluate the deviation from the guidelines to appreciate the magnitude (frequency of lags) and direction of deviation (e.g., whether outlier or systematic deviation). Nonetheless, such effort could also support the identification of best practices: a commonly adopted approach in the healthcare sector that focuses on positive deviance and looks at performance and process variations that lead to positive results [73]. Such a process, in fact, constitutes the real engine of the Learning from Excellence model [74]. The opportunity to support a positive deviance approach via process mining should be evaluated in order to model and regularly spread excellent performances throughout healthcare organizations. In this view, the application of process mining to discover the process actually in use for the care of patients can also support the education of new physicians for specific disease treatments or diagnostic procedures. In the future, the study can be extended to evaluate how much inefficiency in the consumption of resources is linked to deviation from the clinical guideline. From a management point of view, process mining can support studies about the reallocation of resources from inefficient processes, for example, where service duplications are frequent, to high-value process nodes where a more fluid process would ease the care pathway.

## Abbreviations